# Nineteen Whole-Genome Assemblies of *Yersinia pestis* subsp. *microtus*, Including Representatives of Biovars caucasica, talassica, hissarica, altaica, xilingolensis, and ulegeica

**DOI:** 10.1128/genomeA.01342-15

**Published:** 2015-12-03

**Authors:** Angelina A. Kislichkina, Aleksandr G. Bogun, Lidiya A. Kadnikova, Nadezhda V. Maiskaya, Mikhail E. Platonov, Nikolai V. Anisimov, Elena V. Galkina, Svetlana V. Dentovskaya, Andrey P. Anisimov

**Affiliations:** State Research Center for Applied Microbiology and Biotechnology, Obolensk, Russia

## Abstract

The etiologic agent of plague, *Yersinia pestis*, includes two subspecies, of which *Y. pestis* subsp. *microtus* contains the strains that cause only occasional diseases in humans that are not accompanied by human-to-human transmission. Here, we report the draft genome sequences of 19 *Y. pestis* strains (across 6 biovars of *Y. pestis* subsp. *microtus*).

## GENOME ANNOUNCEMENT

The etiologic agent of plague, bacterial species *Yersinia pestis*, includes several phylogenetic groups (1–4). Some of them that display “universal” hypervirulence for different mammal species (epidemic strains or *Y. pestis* subsp. *pestis*) were responsible for three devastating pandemics, while endemic strains circulating in populations of rodents belonging to the genus *Microtus* (*Y. pestis* subsp. *microtus*) were characterized by high virulence to their main hosts, voles, and laboratory mice but as a rule were of low virulence or avirulent for guinea pigs and caused only occasional diseases in humans that were not accompanied by outbreaks of human-to-human transmission of infection (5). The availability of these two closely related *Y. pestis* subspecies that differ in their selective virulence (host specificity) makes it possible to identify by comparing their whole-genome sequences the pathogenicity-determining genes, potential molecular targets for prophylaxis, and treatment of plague.

In this study, we sequenced 19 *Y. pestis* subsp. *microtus* strains (from six biovars: ulegeica, caucasica, xilingolensis, hissarica, talassica, altaica) isolated from different foci.

Whole-genome sequencing was performed using the Ion Torrent PGM (Life Technologies, USA), according to the manufacturer’s instructions. Ion PGM Reagents 400 Kit (Life Technologies, USA) and Ion 318 Chip Kit (Life Technologies, USA) were used for sequencing. For each genome, reads were *de novo* assembled using 2.9 Newbler assembler (Roche). Finally, we obtained from 180 to 341 contigs for each genome (Table 1). The genome size ranged from 4.51 to 4.64 Mb. Each genome contains 3,711 to 4,008 coding sequences. Only 7 strains have all three plasmids (pMT, pCD, pPCP).

**TABLE 1:** Strain-identifying information and basic statistics on assemblies and annotations

Strain name	Alternate strain name	Focus^a^	Raw data accession no.	Assembly accession no.	Size (bp)	No. of contigs	No. of CDS^b^	No. of genes	Plasmid^c^		
									pMT/ pFra	pCD/ pYV	pPCP/ pPst
*Y. pestis* subsp. *microtus* bv. caucasica											
SCPM-O-B-6904	С-537	39	SRR2094286, SRR2094287	LIYP00000000	4,510,568	190	3,916	4,203	+	+	−
SCPM-O-B-7761	C-590	4	SRR2094294	LIYQ00000000	4,560,303	207	4,008	4,249	+	+	−
SCPM-O-B-6990	С-290	4	SRR2094306	LIYU00000000	4,561,770	180	3,952	4,245	+	+	−
SCPM-O-B-6974	С-197	6	SRR2124156, SRR2124157	LIYX00000000	4,522,840	270	3,916	4,255	+	+	−
SCPM-O-B-6757	С-235	7	SRR2124162, SRR2124163	LIYY00000000	4,554,747	228	3,956	4,253	+	+	−
SCPM-O-B-6984	С-267	6	SRR2124165, SRR2124167	LIYZ00000000	4,555,511	231	3,864	4,251	+	+	−
SCPM-O-B-7832	С-359	5	SRR2124169, SRR2124170	LIZB00000000	4,557,841	205	3,854	4,265	+	+	−
SCPM-O-B-6300	С-291	7	SRR2124185	LIZC00000000	4,559,661	202	3,893	4,250	+	+	−
SCPM-O-B-6536	С-346	4	SRR2124186	LIZE00000000	4,560,069	204	3,931	4,243	+	+	−
SCPM-O-B-6540	С-666	5	SRR2124207	LIZF00000000	4,559,559	183	3,863	4,247	+	+	−
*Y. pestis* subsp. *microtus* bv. talassica											
SCPM-O-B-7019	А-1804	40	SRR2124154, SRR2124155	LIYW00000000	4,626,244	275	3,984	4,308	+	+	+
SCPM-O-B-7074	А-1807	40	SRR2094300	LIYT00000000	4,565,589	207	3,966	4,237	+	+	+
*Y. pestis* subsp. *microtus* bv. altaica											
SCPM-O-B-7812	I-3455	36	SRR2094311	LIYV00000000	4,575,487	223	3,939	4,261	+	+	+
SCPM-O-B-7075	А-513	36	SRR2124168	LIZA00000000	4,595,298	229	3,923	4,263	+	+	+
*Y. pestis* subsp. *microtus* bv. ulegeica											
SCPM-O-B-6706	I-3189	M13	SRR2093957	LIYO00000000	4,636,832	189	3,969	4,281	+	+	+
SCPM-O-B-6906	I-2422	M02	SRR2124208, SRR2124209	LIZG00000000	4,520,537	341	3,711	4,212	−	+	+
SCPM-O-B-6213	I-2239	M01	SRR2511857	LIZD00000000	4,636,905	189	3,979	4,288	+	+	+
*Y. pestis* subsp. *microtus* bv. xilingolensis											
SCPM-O-B-6216	I-3134	M20	SRR2094295	LIYR00000000	4,587,862	230	3,920	4,255	+	+	+
*Y. pestis* subsp. *microtus* bv. hissarica											
SCPM-O-B-6304	5307-Gis	34	SRR2094296	LIYS00000000	4,531,080	209	3,847	4,169	+	−	+

a Focus numbers are indicated according to references 1 and 4.

b CDS, coding sequences.

c −, not present; +, present.

The comparative genomic analysis among *Y. pestis* subsp. *microtus* strains under study and other available *Y. pestis* isolates will provide information on the speciation and evolution of *Y. pestis* and might explain the mechanisms of the selective virulence (host specificity) of *Y. pestis* vole strains. A detailed report of a full comparative genomic analysis will be included in a future publication.

### Nucleotide sequence accession numbers.

The GenBank accession numbers for all 19 genomes are listed in Table 1.
